# Investigating pathways linking women’s status and empowerment to skilled attendance at birth in Tanzania: A structural equation modeling approach

**DOI:** 10.1371/journal.pone.0212038

**Published:** 2019-02-13

**Authors:** Kyoko Shimamoto, Jessica D. Gipson

**Affiliations:** Fielding School of Public Health, University of California, Los Angeles (UCLA), California, United States of America; University of Zurich, SWITZERLAND

## Abstract

Maternal and newborn mortality remain unacceptably high in sub-Saharan Africa where use of a skilled birth attendant (SBA) at delivery has remained low. Despite the recognized importance of women’s empowerment as a key determinant of maternal and newborn health, evidence from sub-Saharan Africa is more limited. Using data from the 2010 Tanzania Demographic and Health Survey (n = 4,340), this study employs a robust method–structural equation modeling (SEM)–to investigate the complex and multidimensional pathways through which women’s empowerment affects SBA use. The results show that women’s education and household decision-making are positively associated with SBA use. However, not all empowerment dimensions have similar effects. Attitudes towards sex negotiation and violence as well as early marriage are not significant factors in Tanzania. Mediation analysis also confirms the indirect effect of education on SBA use only through household decision-making. The findings underscore the utility of structural equation modeling when examining complex and multidimensional constructs, such as empowerment, and demonstrate potential causal inference to better inform policy and programmatic recommendations.

## Introduction

Maternal and newborn mortality remain unacceptably high in low- and middle-income countries, where 99.7% of global maternal deaths and 98.5% of newborn deaths occur. Over three hundred thousand women died from causes related to pregnancy and childbirth globally in 2015, and 2.6 million newborns died in 2016, although most of those deaths were preventable [1, 2]. Sub-Saharan Africa has some of the highest rates of maternal and newborn mortality with an average maternal mortality ratio (MMR) of 510 per 100,000 live births and a neonatal mortality rate (NMR) of 28 per 1,000 live births.

The evidence indicates that maternal and newborn deaths could be averted substantially by the professional care provided at childbirth by a skilled birth attendant (SBA) [3]. However, the levels of SBA use remain low, particularly in Africa where only half of deliveries have an SBA present and progress is much slower compared to other regions [4, 5]. The WHO defined an SBA as “an accredited health professional who is proficient in the skills needed to manage normal (uncomplicated) pregnancies and childbirth, and to identify, manage and refer complications in women and newborns” [3]. Investments in healthcare around the time of birth have been identified as the most effective approach to reduce maternal and newborn mortality with an estimated 150,000 maternal lives and 790,000 neonatal lives that could be saved, as well as 550,000 stillbirths that could be prevented by 2025 [3, 6, 7].

Several African countries and other low-income countries struggle to achieve further reductions in maternal and newborn mortality due to a complex set of constraints affecting access to and use of relevant services. Key common barriers include socioeconomic factors (e.g., low education and economic status), geographic factors (e.g., physical distance and transportation means), limited human resources and supplies, and poor quality of care [6, 8–10]. Furthermore, recent evidence and global policy discourse indicate that gender equality and the power women wield in their households, communities and societies are more critical determinants for the health of women and their newborns [6, 10–13]. Although studies from Africa are limited compared to those from Asia, existing studies point to the important influence of women’s status and empowerment, and there is a need for further studies that critically examine the complex and contextual connections between women’s empowerment and maternal health, including delivery care use [8, 12, 14] [15–20].

This study aims to address these needs by employing structural equation modeling (SEM), which is an approach that is useful for investigating potentially complex and multidimensional pathways. We employ SEM to examine the complex pathways between women’s empowerment and SBA use in Tanzania, a setting where more than a third of deliveries are not attended by an SBA.

## Background

### Women’s status, empowerment, and delivery care use

Women’s status and empowerment have been recognized as important influences on a range of health and social behaviors as well as outcomes, including contraceptive use, fertility, delivery care, and, more broadly, poverty reduction and economic growth [11, 13, 21–23]. “Women’s empowerment” is an inherently complex and contextually defined construct, though it is broadly defined as the ways in which women’s social position may affect their abilities to make decisions and to take actions related to their health and well-being [11, 21, 24]. Kabeer defined empowerment as “women’s ability to make strategic life choices”, which comprises three, interrelated dimensions, including “resources, agency, and achievements” [21].

Much of the existing literature assesses “agency” and “resources” through proxy measures, such as women’s participation in household decision-making, access to and control over household resources, and attitudes towards gender norms (e.g., violence, gender equality and relationship) [11, 21, 22]. “Achievements” are the outcomes of empowerment and conceptualized as the ability to make “strategic life choices”, including marriage and childbearing [21]. This domain is increasingly recognized as an important marker of women’s status and empowerment in that the timing and circumstances surrounding these events reflect the extent to which women have a choice in these decisions [25–28]. “Women’s status”, on the other hand, is generally considered to be a measure of “women’s overall position in the society” [24], which is often represented by a woman’s educational attainment as well as her economic or employment status.

Most of the evidence linking women’s status and empowerment with maternal health care use is from South Asia; however, an increasing number of studies are emerging from Africa. One set of African studies examines the influence of education on delivery care use and found generally consistent and positive relationships [14–20, 29–34]. Furthermore, studies that focus more explicitly on the relationships between women’s empowerment and delivery care use in Africa are even more limited and the evidence is mixed [14–20, 29]. Some of these studies examined multiple dimensions of empowerment, finding inconsistent effects of empowerment dimensions on delivery care use [15–18, 20]. For example, a study in eight African countries, including East African countries (e.g., Uganda, Zambia), showed disparate influences of women’s empowerment domains (i.e., decision-making, attitudes towards gender norms) on delivery care [15]. A subsequent study using multi-level modeling that controlled for non-independence within the country found no significant effect of household decision-making on delivery care use [17]. Moreover, despite growing concerns regarding the connections between early marriage, childbearing and women’s empowerment [35–37], studies are very limited in Africa compared to Asia. Only one cross-national study was identified that examined this issue in Sub-Saharan Africa, demonstrating that early childbearing adversely influences women’s future empowerment [25].

### Methodological approaches

The disparate relationships between women’s empowerment and maternal health may be explained, at least in part, by two key methodological issues. First, there is no standard operationalization or measurement of empowerment, which makes the study of empowerment inconsistent across studies and settings [11, 22]. The importance of considering the multiple dimensions of empowerment [21] has been increasingly stated, yet summative measures or composite indices are often employed, which results in an inability to deconstruct the distinct influence of each empowerment dimension. Several studies have incorporated multiple empowerment dimensions [15–20, 28]; however only a handful of studies have employed analyses (e.g., factor analyses) to specifically examine the underlying structures and relationships between empowerment domains when examining maternal and reproductive health outcomes [20, 29, 36, 38–41].

Second, the existing global studies on delivery care have rarely employed SEM, despite its usefulness in identifying the complex connections between distinct empowerment domains and health outcomes. The current manuscript builds on work conducted by the authors in two, separate analyses [20, 41]. In the first analysis, authors assessed SBA use in Senegal and Tanzania using simple multivariate analyses. A subsequent analysis employed SEM to examine the complex mechanisms through which the multiple empowerment domains affect SBA use specifically in Senegal [41], and to provide greater potential into causality of these pathways [42, 43].

## Conceptual framework

**Fig 1:**
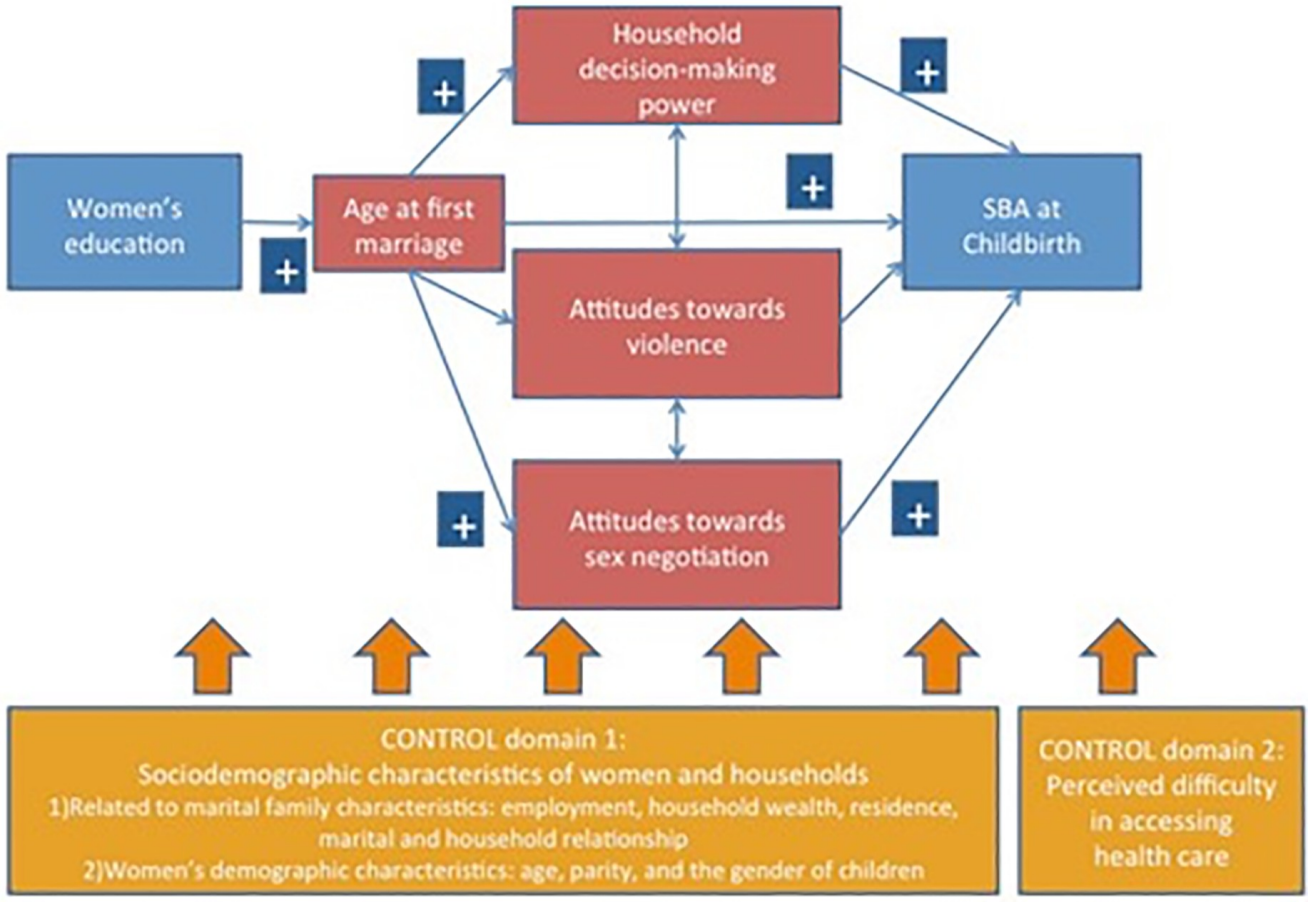
An integrated conceptual framework to predict influences of women’s status and multi-dimensional empowerment on SBA use.

This analysis employed an integrated conceptual framework from the Theory of Gender Stratification [44, 45] to identify the social determinants to women’s reluctance or inability to seek reproductive health services, which results in negative health outcomes such as maternal mortality (Fig 1). Fig 1 shows that women’s status, which was operationalized as women’s education, promotes empowerment, which in turn increases SBA use. Specifically, the theory and framework would suggest that the greater women’s power is, the more they have “control over their lives” and various “life options” (e.g., marriage, divorce, and sexuality) [44].

The relationship between education and SBA use is hypothesized to be mediated by multiple dimensions of empowerment. Specifically, a woman with more education is likely to marry later, to have higher household decision-making power and to have more progressive attitudes towards gender norms. Consequently, women with more education are more likely to use an SBA. These empowerment dimensions are frequently chosen and examined in studies using the Demographic and Health Survey (DHS) data [27]. Furthermore, this framework considers the potential confounding effects of sociodemographic characteristics of women and households as well as the perceived difficulty in accessing health care [10].

## Methods

### Study setting and data

Maternal and neonatal mortality has declined in Tanzania over the last two decades (MMR from 910 to 410; NMR from 41 to 22); however, the country ranks in the top ten countries in the world in terms of the number of annual maternal deaths (8,200) [1, 2]. Maternal and newborn mortality reductions are likely contingent on increasing SBA use during childbirth. According to the most recent surveys, 64% of deliveries were attended by SBAs in 2015, which was an increase from 51% in 2010 [46, 47].

Examining the connections between women’s empowerment and SBA use is particularly important in Tanzania given the recent shifts in gender relations and their countervailing influences on maternal health. Studies from Tanzania and other eastern African countries suggest that women’s status and power in households is increasing alongside notable declines in patriarchal marital customs (e.g., arranged marriage) [48, 49]. Other norms and traditional customs (e.g., early marriage, sexual debut), however, appear to be unchanged[46].

This study used data from the 2010 Tanzania DHS, which is a nationally representative household survey [46]. The study sample consisted of currently married women who had at least one birth during the five years preceding the survey. Questions on household decision-making were asked of married women only; therefore, unmarried women were removed from the analysis. Furthermore, women with missing data concerning decision-making, gender norms, and sociodemographic variables were removed, which resulted in a final analytic sample of 4,340 women (weighted). The proportion of missing observations is marginal (only 3.7 percent), and thus, potential bias due to missing data is negligible. All analyses included household and individual weights to adjust for differences in the probability of selection and interviews among the individuals included in the sample. In the DHS surveys, trained interviewers provided a standard introduction at the beginning of the interview to explain the study and to seek consent for participation. The interview was undertaken with participants who provided verbal consent. This was a secondary data analysis of the global survey for which ethical approvals were already obtained by an ICF Institutional Review Board (IRB) and an IRB in the host country. Thus, a separate ethical approval for this specific study was not required by the UCLA IRB.

### Analytic strategy and measures

Latent variable SEM was employed for this study, which included both a structural portion (i.e., with measured variables) and a measurement portion (i.e., with latent constructs). The latent variable SEM was comprised of three latent constructs, each of which included individual empowerment indicators to represent each dimension. The latent variable SEM included five endogenous variables (i.e., variables that appear as dependent variables in at least one equation) and multiple exogenous variables (i.e., variables that are never dependent variables). The endogenous variables were SBA use, household decision-making, gender-role attitudes and age at first marriage. The exogenous variables were women’s education, sociodemographic characteristics of women and households and perceived difficulty in accessing health care.

#### Endogenous variables

***SBA use*** at childbirth was operationalized as the use of SBA(s) for the last childbirth. The variable was recoded as binary per the WHO definition [3]. SBAs included a doctor or an assistant medical officer, a clinical officer, a nurse or a midwife. Non-SBAs included an MCH aide, a village health worker, a traditional birth attendant, a relative or friend, another individual, or an unattended delivery.

***Age at first marriage*** was examined as a continuous variable that was calculated by MEASURE DHS, and it was based on the date of the first marriage or union (“living with a man as if married”) and a respondent’s date of birth.

***Household decision-making power*** was examined as a latent construct consisting of three indicators: women’s participation in decisions regarding their own health care, major household purchases, and visits to family/relatives. The variables were first recoded to examine if women participated in decisions (i.e., alone or jointly with their husband) or not. A latent variable was constructed from the three binary variables for each decision.

***Attitudes towards violence*** was examined as a latent construct consisting of five indicators: women’s acceptance of wife-beating by her husband/partner under five situations, including if she goes out without telling him, neglects the children, argues with him, refuses to have sex with him, or burns the food. The variables were also recoded as binary (i.e., yes or no).

***Attitudes towards sex negotiation*** were also assessed as a latent construct based on two questions regarding women’s perceived ability to negotiate sexual relations, including if the respondent can refuse to have sex or can ask her husband to use a condom. The variables were recoded to determine if the respondent can refuse/ask or not (i.e., cannot refuse or ask, do not know, not sure, or depends).

#### Exogenous variables–Women’s education and control variables

***Women’s education*** was measured as a continuous variable representing the number of years that the woman attended school. ***Sociodemographic characteristics of women and households*** were included in the model as control variables. The characteristics included a *woman’s age*, *parity*, *paid employment*, *household wealth*, *marital and household relationship*, *gender of children*, *place of residence*, *and a woman’s age and education relative to her husband*. *A woman’s age* at delivery and parity (i.e., the birth order of the child) were examined as continuous variables based on preliminary analyses indicating linear relationships with SBA use. *Paid employment* was defined as a woman who had been employed for cash or in-kind in the last 12 months or not employed as a binary measure. *Household wealth* was determined based on reported ownership of household assets (e.g., consumer items and home attributes). A composite index was created by the DHS MEASURE that was based on the results of the principal component analysis and divided into quintiles [46]. *Marital relationship* was assessed as categorical, including monogamous union, polygamous as a first wife, or polygamous as a second wife or lower, to consider differences according to the type of relationship and wife order given evidence of differences in women’s status and power across these relationship types [48–50]. *Household relationship*, which was a binary variable, indicated if the respondent was a household head or not. The *genders of children* was a binary measure to assess if the respondent had at least one living son or not at the time of the delivery, as having a son has been shown to be an important reflection of African women’s status and power [51]. The *place of residence* was a binary measure and indicated whether the respondent lived in an urban or rural area. *Women’s age relative to husbands* was assessed as continuous, whereas relative education was examined as a categorical variable.

***Perceived difficulty in accessing health care*** was also included as a control variable and included items such as getting permission to go, getting the money needed for advice or treatment, distance to a health facility, or not wanting to go alone. The variables were first recoded into binary variables to demonstrate whether the respondent perceived a big problem or not (i.e., not a big problem or not a problem at all). A continuous variable showed the number of aspects for which the respondent perceived difficulties (scored 0–4).

### Analytical models and steps

Data analysis was conducted in three main steps. First, descriptive analyses were conducted using SAS 9.3. Second, factor analyses were conducted using Mplus version 7.3 with a geomin rotation. Exploratory factor analysis (EFA) identified the number of factors/latent constructs and the underlying factor structure of empowerment. Consequently, a three-factor confirmatory factor analysis (CFA) examined the appropriateness and generalizability of the measurement portion of the SEM through the examination of the fit statistics of the portion and the statistical significance of path coefficients using t-values [43]. In the CFA, the path of the first indicator is constrained to 1 (thus t value was not calculated), and the significance of t-values refers to unstandardized parameter values. Third, a SEM was conducted with Mplus using polychoric correlations for categorical variables to examine the pathways from women’s education to SBA use. All the analyses were conducted while accounting for individual weights, clusters (i.e., primary sampling unit), and sample strata using the survey analysis commands.

The SEM models analyzed five equations simultaneously for all five endogenous variables in the model and estimated standardized coefficients; thus, the SEM also provided an examination of the multidimensionality of empowerment and the relative importance of one dimension over others. These equations separately regressed SBA use, household decision-making power, attitudes towards violence, attitudes towards sex negotiation, and age at first marriage using probit regression with weighted least squares (WLSMV) for more precise coefficient estimates than logistic regression, especially for mediation analysis [52]. This SEM employed listwise deletion with missing observations due to the negligible proportion of missing observations in the study sample.

In the model, all of the exogenous variables were designated as covarying due to the potential relatedness among exogenous variables. Additionally, the errors/disturbances of empowerment dimensions were all covarying due to the possible relatedness among the unobserved aspects of these constructs.

Model fit was assessed using recommended model fit indices, including root mean square error of approximation (RMSEA) < 0.06 (or less as a “close” fit) and a comparative fit index (CFI)/ Tucker-Lewis index (TLI) with ≥ 0.95 [53]. Weighted root mean square residual (WRMR) (less than 0.90) was also calculated by Mplus for the models with categorical endogenous variables [54].

## Results

### Descriptive results

Half of the respondents used an SBA at their most recent birth (Table 1). On average, women scored in the mid-range of the scales, indicating moderate levels of household decision-making and gender norm attitudes. Women got married or started a union at a mean age of 18.3 years, and they had an average of five years of education. On average, these women gave birth around four children, and two in five women were employed for cash payment. The majority of these women lived in rural areas and were in a monogamous union (79% respectively).

**Table 1:** Characteristics of participating, currently married women with at least one birth in last 5 years (n = 4,340 weighted; n = 4,334 unweighted), TDHS 2010.

			Freq	Weighted	
Variables				Mean or Proportion	SE
**Outcome**					
	Skilled Birth Attendant use at the last childbirth		2,190	50.74	1.52
**Mediators—Women's empowerment measures**					
	Household decision-making (Mean, scored 0–3)			1.43	0.02
** **	Attitudes towards violence (Mean, 0–5)			3.15	0.04
** **	Attitudes towards sex negotiation (Mean, 0–2)			1.38	0.02
	Age at first marriage (Mean)			18.28	0.07
**Demographics and perceived accessibility of health care**					
	Education (Mean in years)			5.07	0.09
	Current age			29.41	0.15
	Household wealth quintile				
		Poorest	809	19.77	1.08
		Poorer	944	22.80	0.98
		Middle	889	21.40	0.92
		Richer	936	19.93	1.13
		Richest	756	16.09	1.17
	Employment for payment				
		Currently employed or employed last 12 months	1,688	37.96	1.09
	Parity (Total number of children ever born)			3.92	0.05
	Marital relationships				
		Monogamous union	3,364	79.09	1.05
		Polygamous as 1st wife	428	8.86	0.52
		Polygamous as 2nd or lower	542	12.05	0.80
	Household head		247	5.76	0.48
	Place of residence				
		Urban	855	21.31	1.19
		Rural	3,479	78.69	1.19
	Having son(s)		3,573	81.54	0.66
	Relative age			-7.21	0.15
	Relative education				
		More education than husband	867	17.43	0.68
		Almost the same (the same, or slightly less within 2 years)	2,154	52.76	1.04
		Less education than husband (by 2 years or more)	1,313	29.81	1.02
** **	Perceived difficulty of health care (Mean, scored 0–4)			0.61	0.02

Note: Frequency missing = 1 (with decision-making), 82 (with perceptions of gender norms against violence), 4 (with employment), 32 (with marital relationships), 23 (with relative age), and 17 (with perceived difficulty in accessing health care).

### Factor analysis results

The exploratory factor analysis results defined three factors as follows (Eigenvalues >1.0) (Pett, 2003): 1) “***household decision-making power***” (three indicators); 2) “***attitudes towards violence***” (five indicators); and 3) “***attitudes towards sex negotiation****”* (two indicators) (See Table 2). The three factors were shown to be significantly correlated, albeit not very highly, with correlation less than 0.25 (p<0.05), which suggests that each factor is distinct. The three factor confirmatory factor analysis results including fit statistics and t-values support the appropriateness and generalizability of the measurement portion of the SEM (See Table 2) (p<0.001).

**Table 2:** Factor analysis for indicators of empowerment (n = 4,340), TDHS 2010.

*Latent construct*	*Aspects that survey asked*	*Factor loadings (EFA)*	*t value (CFA)*
Household decision-making	Decision on own health care	0.795	-
	Decision on major household purchases	0.873	32.338
	Decision on visits to family or relatives	0.933	32.268
Attitudes towards violence	Violence if going out without telling her husband	0.887	-
	Violence if neglects the children	0.922	84.620
	Violence if argues with him	0.933	84.459
	Violence if refuses to have sex with him	0.881	79.617
	Violence if burns the food	0.866	56.159
Attitudes towards sex negotiation	Perceived ability in refusing sex	0.894	-
	Perceived ability in asking condom use	0.658	8.059

Note: All factor loadings are significant at p<0.05. RMSEA = 0.028 (CI = 0.024–0.033); CFI = 0.995; TLI = 0.993.

### SEM results

**Fig 2:**
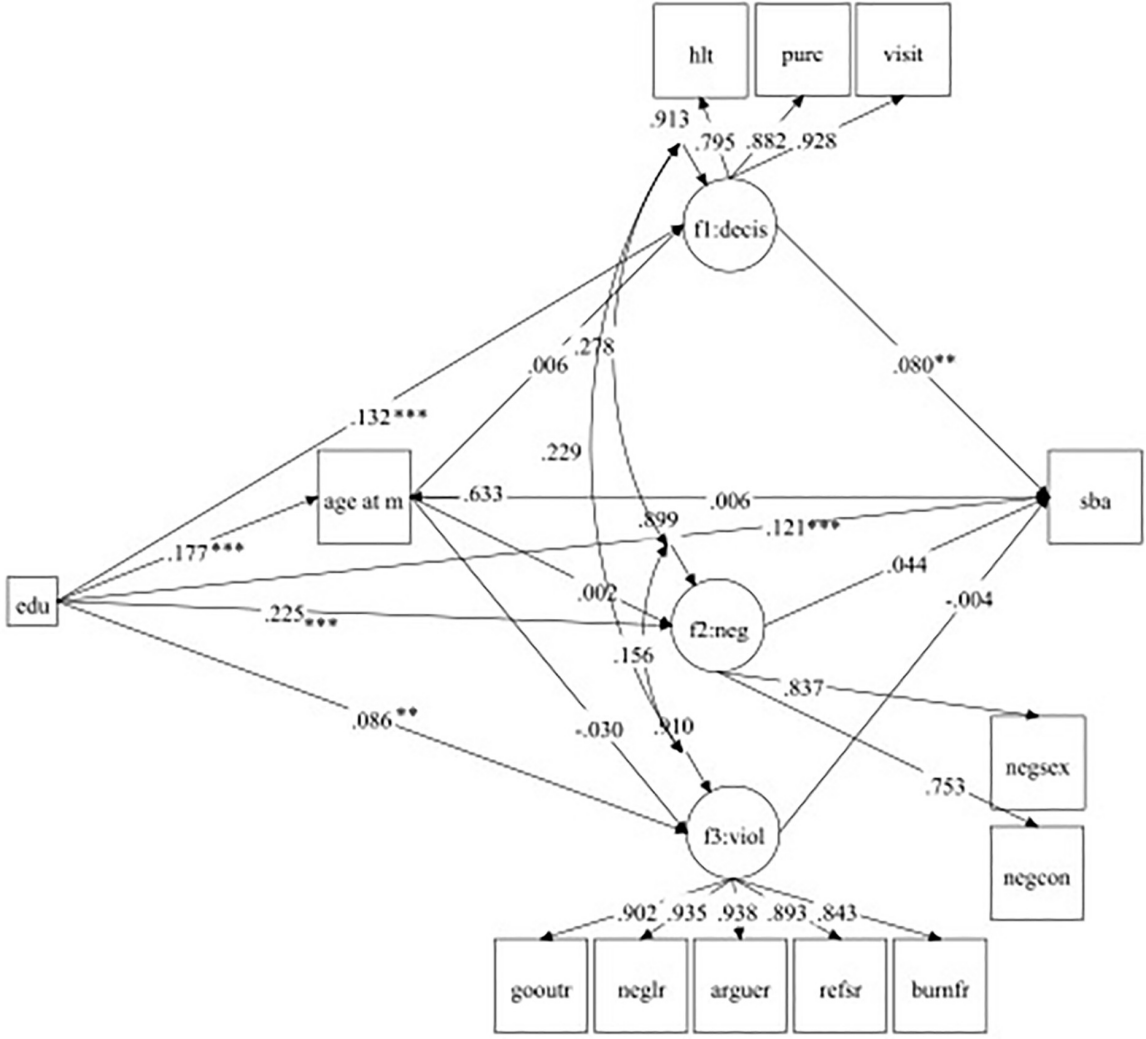
A diagram of the latent variable SEM. Note: sba = SBA use; f1:decis = decision-making power; f2:neg = attitudes towards sex negotiation; f3:viol = attitudes towards violence; age at m = age at first marriage; edu = education. Standardized path coefficients are reported with the following significance levels: *** p <0.001; ** p<0.01; * p<0.05. Factor loadings and correlations among disturbances are all significant at p<0.001. Control variables are not included in the figure.

The final adjusted SEM results are presented in Table 3 and Fig 2. The path coefficients are standardized, while the p-values are reported in the unstandardized metric by default. The model fit statistics (RMSEA, CFI/TLI, WRMR) support that the models fit the data well.

**Table 3:** Standardized path coefficients of the latent variable SEM (n = 4,340 for the analysis), TDHS 2010.

		Dependent variables in the equation (Y):				
Predictors in the equation (X):		[Column 1] Age at first marriage	[Column 2] Decision-making power	[Column 3] Attitudes towards violence	[Column 4] Attitudes towards sex negotiation	[Column 5] SBA use
	Endogenous variables					
	(1) Age at first marriage		0.006	-0.030	0.002	0.006
	(2) Decision-making power					0.080**
	(3) Attitudes towards violence					-0.004
	(4) Attitudes towards sex negotiation					0.044
	Exogenous variables					
	Education	0.177***	0.132***	0.086**	0.225***	0.121***
	Age at childbirth	0.908***	0.194***	0.136**	0.013	0.181***
	Parity	-0.804***	-0.111*	-0.132**	-0.045	-0.211***
	Employment for payment	-0.015	0.062**	0.083***	0.055*	0.046
	Household head	0.003	0.099***	-0.019	0.068**	0.005
	Urban residence	0.013	0.031	-0.047	0.030	0.152***
	Having son(s)	-0.051**	0.055*	0.002	0.006	-0.070**
	Household wealth (the 2nd lowest)	0.017	-0.033	0.065*	0.020	0.014
	Household wealth (the 3rd lowest)	0.010	-0.050	0.000	0.034	0.080**
	Household wealth (the 4th lowest)	-0.026	-0.091*	0.039	0.039	0.150***
	Household wealth (the highest)	0.002	0.006	0.239***	0.084	0.289***
	Polygamous union as a first wife	-0.057**	-0.060**	-0.023	-0.018	-0.061**
	Polygamous union as a second or lower	0.036*	-0.117***	-0.001	-0.069**	-0.065**
	Relative age	0.064***	0.036	0.022	0.011	0.001
	Less education than husband	0.062**	0.032	0.047	0.052	0.038
	More education than husband	0.031	-0.025	0.007	0.033	-0.032
	Perceived difficulty in accessing health care					-0.121***

Note

***p <0.001

**p<0.01

*p<0.05.

Reference groups: residence = rural; household wealth = the lowest; marital relationship = monogamous union; relative education = almost the same education with husbands. DF = 169, RMSEA = 0.013, CFI = 0.993, TLI = 0.989, WRMR = 0.849. R-square: 0.367 (age at first marriage), 0.087 (decision-making), 0.090 (perceptions against violence), 0.101 (perceptions for sex negotiation), 0.395 (SBA use).

Women’s education and household decision-making power were positively related to SBA use (Column 5 in Table 3) (i.e., the standardized coefficient for education = 0.121 and decision-making = 0.080)]. However, age at first marriage and perceptions of gender norms were not significant predictors of SBA use.

Additionally, age, urban residence, and higher household wealth were positively related to SBA use. Parity, having living son(s), being in a polygamous union, and perceived difficulties in accessing health care were negatively associated with SBA use. Education was positively related to age at first marriage (b = 0.177, Column 1 in Table 3). Other significant and positive covariates for predicting age at first marriage included women’s older age, being the second or lower rank wife in a polygamous union, and a husband’s higher education relative to women. However, parity, having son(s), and being the first wife in a polygamous union were negatively related to age at first marriage.

Education was positively associated with household decision-making power (b = 0.132), progressive attitudes towards violence (b = 0.086), and sex negotiation (b = 0.225) (Column 2–4, Table 3). As shown in Column 2, higher education, older age, paid employment, being head of the household and having son(s) were positively related to decision-making, whereas higher parity and polygamous relationships were negatively related.

In Column 3 (Table 3), significant covariates in predicting attitudes towards violence included higher education, older age, lower parity, paid employment, and a lower wealth quintile. Additionally, higher education, paid employment and being head of the household were positively related to progressive attitudes towards sex negotiation, whereas a polygamous marital relationship as a second or lower wife order was negatively related (Column 4).

The direct and indirect effects of women’s education on SBA use are shown in Table 4. The total indirect effects of education on SBA use were significant via the identified multiple pathways. However, only the specific indirect effect through household decision-making power was significant. This outcome suggests that women’s higher education positively affected decision-making power, which in turn increased SBA use. This specific indirect effect of education (b = 0.011) contributes to the majority of the indirect effect with a substantial proportion of the total indirect effect relative to the direct effect (17.4%).

**Table 4:** Summary of standardized direct and indirect effects of education on SBA use (n = 4,340), TDHS 2010.

		coefficient	t value
Total effect		0.143	5.212***
Direct effect		0.121	4.326***
Total indirect effect		0.021	2.430*
Indirect effect via	Age at first marriage	0.001	0.220
	Decision-making power	0.011	2.335*
	Attitudes towards violence	0.000	-0.144
	Attitudes towards sex negotiation	0.010	1.239
	Age at first marriage then decision-making power	0.000	0.256
	Age at first marriage then attitudes towards violence	0.000	0.070
	Age at first marriage then attitudes towards sex negotiation	0.000	0.145

Note

***p <0.001

**p<0.01

*p<0.05.

## Discussion

This study investigated the mechanism through which women’s status and empowerment affect SBA use during childbirth in Tanzania. The findings demonstrated evidence of the multiple and sequential effects of women’s status and empowerment, such as direct, indirect and mediating effects. This evidence underscores the contribution of education on SBA use through multiple and sequential empowerment pathways and demonstrates the potential causal mechanisms influencing SBA use. This study also confirmed the multidimensional and contextual nature of empowerment as it relates to maternal health care use.

Five key findings arise from this analysis. First, the study showed the significant and positive direct effect of women’s education on age at first marriage, decision-making power, and progressive gender norm attitudes and SBA use. These findings are generally consistent with existing evidence on women’s education and other sociodemographic characteristics as predictors of delivery care use and outcomes [8, 14–20, 29–33].

As hypothesized per the integrated conceptual framework, the effects of education on delivery care use were mediated through women’s decision-making power. The positive influences of education and household decision-making power on SBA use align with some of the existing global evidence that women’s status and decision-making power are a key determinant of delivery care use as well as other reproductive health behaviors and outcomes [6, 10–12, 22]. These specific direct and indirect pathways were examined in a previous study conducted in Senegal by the authors; however, the significant dimensions of empowerment differed in Senegal such that attitudes towards gender norms were a significant mediator, instead of decision-making power as shown in this analysis in Tanzania. [41]. Both studies suggest potential causal pathways through which education may positively influence some empowerment domains and subsequent delivery care use. This evidence calls for policy makers and practitioners to further invest in women’s education for the dual purpose of empowering women for gender equality and of positively influencing maternal health outcomes [4, 35, 55, 56].

Second, the significant mediating effects of decision-making power on SBA use also highlight the importance of this construct in measuring women’s empowerment in general and for examining the shifts in household structure in Tanzania from predominantly extended to nuclear households [48, 49]. With an increasing proportion of households consisting of nuclear families in Tanzania and other global settings [48, 49, 57], women’s ability to participate in decisions becomes an increasingly important marker of their continued, or perhaps even expanded, engagement in decisions in the household amidst these broader social changes.

Third, the factor analysis results stressed that the construct of household decision-making was best represented by all three broader indicators of women’s decision-making power (i.e., own health care, household purchases, visits to family and relatives), and not limited to women’s ability to seek their own health care. The relevance of all three measures in predicting SBA use highlights the importance of looking more holistically at the components of household decision-making and their effects on the process of seeking and obtaining health services, especially in the context of Tanzania and elsewhere, where women’s roles are undergoing a transition. As noted by other scholars, the decisions on health care seeking, particularly in a resource-constrained setting, is complex and contingent upon multiple logistical and structural factors, such as the distance to facility, and the availability and quality of care [6, 10, 13]. Following the earlier advancement of gender perspectives and interventions to transforming gender norms and relationship in other reproductive health programs [58–60], the findings from this study emphasize the importance of maternal health interventions that seek to promote equitable gender relations and decision-making dynamics among couples and in the household.

Fourth, the use of SEM in this analysis provided the simultaneous comparison of several empowerment domains on SBA use, which reveals variations in the magnitude and significance of each domain. Two specific insights garnered from the SEM, including the null effects of age at first marriage and gender norm attitudes on SBA use in terms of both direct and mediating effects, were unexpected. These findings are in contrast to those of other studies that demonstrated significant effects of gender attitudes on SBA use in other African settings [15–17, 20, 41], including the findings from Senegal [20, 41]. These findings may reflect the ongoing transitions in social norms regarding gender relations and marriage in Tanzania [48, 49]. To address the complex nature and mechanisms through which women’s empowerment may influence various reproductive and maternal health outcomes, especially within dynamic social settings, it is critical to generate evidence, especially through implementation research, to enhance women’s status and empowerment in an effort to achieve health and broader development goals [13, 23, 55, 56].

The multidimensionality of empowerment is affirmed by the varied effects of the respective empowerment domains on SBA use; hence, there is a need for multidimensional operationalization and measurement of women’s status and empowerment. Furthermore, given that SEM provides an opportunity to examine these dimensions as latent constructs, it was possible to consider the contribution of each indicator and the respective measurement errors. Indeed, the residuals for these three factors (i.e., empowerment domains) were significantly correlated with each other, which suggests that there may be similar or shared features in the unexplained aspects of these latent variables. SEM is uniquely equipped to address such complexities and to measure and deconstruct intervening effects, although the empirical applications of SEM to such data are still limited in the literature.

Finally, findings from this study both align with and deviate from those of previous studies examining the influences of sociodemographic characteristics on SBA use. For example, consistent with previous reviews and renewed concerns regarding the provision of equitable maternity care [13], household wealth and urban residence were positively associated with SBA use, and perceived difficulty in accessing health care was negatively related to SBA use [6, 10]. On the other hand, the null finding from this study regarding paid employment can be compared to cross-national studies in Africa that have found mixed evidence of women’s employment as a marker of women’s empowerment/status [15, 20, 29], and the results caution against the presumption that employment connotes higher status/power across contexts [61].

Despite the strengths of this study, there are some limitations. First, any causal inference is tentative due to the cross-sectional survey dataset that was employed. Therefore, despite the strength of SEM, the direction of causation remains uncertain due to potential reciprocal effects (e.g., school girls might have dropped out due to marriage or pregnancy; however, the hypothesized direction is supported by relevant theories and descriptive results. In accordance with the definition of empowerment as a *process* in acquiring power, its effect should ideally be examined over time using longitudinal data. Second, the operationalization and measurement of women’s status and empowerment was informed by the data availability in the DHS, while the relevance of empowerment measures has not been highly supported in Africa to a greater extent than in Asia, where the measures were developed [27]. Third, the representativeness of the study sample and generalizability of the results are limited due to the omission of unmarried women, whereas measures of women’s power should ideally capture “strategic life choices” that are relevant regardless of marital status [21, 44]. The omission of young adolescents from the survey respondent is a serious gap given the evidence of risks and burdens carried by adolescents regarding unskilled deliveries, unsafe abortions, and maternal deaths [35, 62–65].

This study, however, is one of the first theory-based studies that uses nationally representative data to investigate complex mechanisms through which women’s status and empowerment affect SBA use during childbirth. This evidence affirms the multi-dimensionality of empowerment and its mediating role in the mechanism leading to professional delivery care. This finding calls for further investment in evidence-based policies and interventions in Tanzania, and possibly in other African countries, to increase education among women, to intensify legal and societal efforts to end child marriage and to facilitate women’s participation in household decision-making in an effort to promote SBA use and to achieve maternal and newborn mortality reductions.

## Abbreviations

DHS: Demographic and Health Surveys
SBA: Skilled Birth Attendant
